# Assessing Alopecia Areata Misinformation: Social Media Analysis

**DOI:** 10.2196/90088

**Published:** 2026-07-23

**Authors:** Shanika N Francis, Ank A Agarwal, Aaron H Fanous, Dawn H Siegel, Roxana Daneshjou

**Affiliations:** 1 Meharry Medical College, School of Medicine, 1005 Dr DB Todd Jr Blvd., Nashville, TN, 37208, United States, 1 (615) 327-6000; 2 Department of Biomedical Data Science, Stanford University, 1265 Welch Rd, Stanford, CA, United States; 3 Department of Dermatology, Stanford University, Stanford, CA, United States

**Keywords:** alopecia areata, social media, digital health, health literacy, health communication, patient education

## Abstract

**Background:**

Alopecia areata (AA) is an autoimmune, hair loss–inducing disease affecting individuals of all genders and ethnic backgrounds worldwide. As social media platforms and artificial intelligence chatbots increasingly influence patient behavior, individuals with AA may turn to these sources for treatment guidance.

**Objective:**

This study evaluated the accuracy of AA-related information on social media and assessed whether patients were exposed to misinformation that could hinder appropriate care. Influencers may recommend treatments applicable to alopecia broadly or promote therapies lacking medical benefit.

**Methods:**

YouTube and TikTok were searched using the hashtags #alopecia and #alopeciatreatment. Posts were selected based on hashtag relevance. Videos were categorized using standard AA treatment guidelines into (1) accurate and medical data–backed (aligned with guideline-supported treatments), (2) limited or anecdotal evidence (unverified treatments without clear misinformation), (3) misinformed and/or false (inaccurate or misleading claims), or (4) other (unrelated to alopecia management).

**Results:**

A total of 98 YouTube and 222 TikTok videos related to #alopecia and #alopeciatreatment were analyzed. Of the YouTube videos analyzed, 76% (n=75) were categorized as “other,” 13% (n=13) as “accurate and medical data–backed,” and 10% (n=10) as “limited or anecdotal evidence”; no videos were classified as “misinformed and/or false.” Of the TikTok videos analyzed, 76% (n=170) were categorized as “other,” 17% (n=38) as “accurate and medical data–backed,” 5% (n=12) as “limited or anecdotal evidence,” and 1% (n=2) as “misinformed and/or false.” Among videos specifically discussing AA treatment, 56% of YouTube videos and 73%of TikTok videos were categorized as “accurate and medical data–backed.” The difference in accurate vs limited or nonmedical YouTube content was statistically significant (*P*=.002), while misinformation prevalence between platforms was not significantly different (*P*=.15).

**Conclusions:**

Patients with AA are frequently exposed to generalized alopecia content on social media, which may not consistently offer disease-specific guidance. Health care professionals should help ensure that social media platforms and artificial intelligence tools are leveraged to promote accurate education and proactively combat misinformation surrounding AA treatment.

## Introduction

Alopecia areata (AA) is an autoimmune disease prevalent in 2% of the worldwide population that affects both men and women from a range of ethnic backgrounds [1]. AA also affects 1 in 1000 children and adolescents [2]. The physical presentation of AA includes noncicatricial (nonscarring) hair loss—which maintains the potential for regrowth and does not degrade the hair follicle [3]. The disease is also highly impactful on the psychosocial well-being of diagnosed patients; due to negative public associations of hair loss with poor health, many patients with AA experience social discrimination, impairment, and stigma [4].

Social media platforms have become vital components in shaping patient behaviors and trends, especially related to alopecia [5]. Social media influencers use platforms such as Instagram, TikTok, and YouTube to disseminate treatment recommendations to a broad audience, including young adults and children [6]. When experiencing hair loss, patients tend to seek medical advice from social media influencers instead of their health care provider or prior to their first consultation [1]. However, concerns have emerged regarding the accuracy and safety of alopecia-related advice provided by influencers, especially when compared to recommendations from dermatologists [5]. This study aimed to review the accuracy of the information about AA on 2 video-based social media platforms, YouTube and TikTok, and investigate whether patients may potentially receive misinformation that could either deter or impair appropriate AA treatment from their health care providers [5].

Understanding the information on social media about AA treatment will be critical for guiding physicians on how to better proactively inform their patients with accurate treatment information and to create effective educational materials for correctly managing the disease.

## Methods

### YouTube and TikTok Data Collection

A web scraping platform, Apify (Apify Technologies sro), was used to extract alopecia-related videos’ data from YouTube and TikTok. To identify relevant posts, we selected videos with hashtags #alopecia or #alopeciatreatment in the video caption, description, or title. A total of 98 and 222 posts were collected from YouTube and TikTok respectively between July 17, 2024, and July 24, 2024. Videos retrieved through hashtag searches were manually screened for relevance by 1 reviewer (SF) prior to application of the inclusion and exclusion criteria. Duplicate or reposted videos were excluded from analysis when identified. Included posts were required to meet the following inclusion criteria: (1) posted by dermatologists who explicitly stated their title or had their credentials listed vs other users and (2) focused on hair loss treatment discussions or advice.

For criterion 1, dermatologist status was identified based on self-reported professional titles or board-certified medical doctor credentials included within the caption, title, profile, or video content.

To ensure standardized creator classification, only self-identified US-trained board-certified dermatologists with medical doctor credentials were included. Posts from creators who did not identify as dermatologists were included in the analysis. For criterion 2, posts were excluded if they were unrelated to AA (eg, traction alopecia) or if they were advertisements or non-English content. The relevancy of the video to various hashtags was confirmed by examining post captions for the searched hashtags and relatedness of the content to alopecia to ensure appropriate categorization.

### Content Categorization

Data were manually reviewed and categorized based on whether the treatment information provided in the video aligned with medical standards for treating AA. Posts were grouped into four categories: (1) accurate and medical data–backed, (2) limited or anecdotal evidence, (3) misinformed and/or false, and (4) other. Posts classified as “accurate and medical data–backed” referenced treatments aligned with established American Academy of Dermatology or National Alopecia Areata Foundation guidelines, such as corticosteroid injections, topical minoxidil, or platelet-rich plasma therapy. Posts categorized as “limited or anecdotal evidence” included treatments with limited, inconsistent, or primarily anecdotal clinical support, such as herbal oils, supplements, scalp massage, or alternative therapies. While these treatments were not necessarily directly misleading or false, they were not strongly supported by current evidence-based dermatologic guidelines. Limited or anecdotal evidence and “misinformed and/or false” posts included content that directly contradicted established medical guidelines, promoted unsupported curative claims, or recommended treatments without any supporting evidence.

US Food and Drug Administration–approved therapies for AA were categorized as evidence-based treatments. Off-label therapies were evaluated according to consistency with established dermatologic guidelines and recommendations from the American Academy of Dermatology and the National Alopecia Areata Foundation. Posts categorized as “other” included those focused on types of alopecia not related to AA, advertisements, treatment progress updates, or unrelated topics (eg, wig modeling).

### Statistical Analysis

Data visualization and analysis were performed using Python 3.3.1 (Python Software Foundation) with Matplotlib: descriptive statistics were used to quantify the proportion of posts falling within each categorization group across both platforms. For instance, of the 98 posts on YouTube, 13% (n=13) were categorized as “accurate and medical data–backed,” and 76% (n=75) were classified as “other.” A similar approach was used to analyze TikTok posts. Further statistical analysis, including chi-square tests, was performed to assess the difference in the prevalence of misinformation between YouTube and TikTok posts (*P*<.05 was considered statistically significant). These comparisons were performed to evaluate whether misinformation prevalence differed between platforms and to better understand potential platform-specific patterns in AA treatment content.

### Ethical Considerations

All necessary permissions were obtained for the use of data scraping tools, and content collection adhered to platform policies [7,8]. Given that no personal data were collected and the study involved publicly available information, institutional review board approval was not required per Stanford University guidelines [9].

## Results

### YouTube Content Analysis

A total of 98 YouTube videos related to #alopecia and #alopeciatreatment were identified and analyzed. Of these, 76% (n=75) of videos were categorized as “other,” as they did not address AA treatment specifically. The remaining 26% (n=23) of videos addressed AA treatment specifically. Among these, 56% (n=13) were categorized as “accurate and medical data–backed,” promoting evidence-based treatments such as corticosteroid injections, platelet-rich plasma therapy, and topical or oral minoxidil (Table 1). The remaining 43% (n=10) were classified as “limited or anecdotal evidence,” with content recommending alternative treatments such as garlic oil, ginger, rosemary, and scalp massages. Some influencers suggested seeing a dermatologist if no improvement was seen after 3 to 4 months of using these alternative treatments.

**Figure 1.**
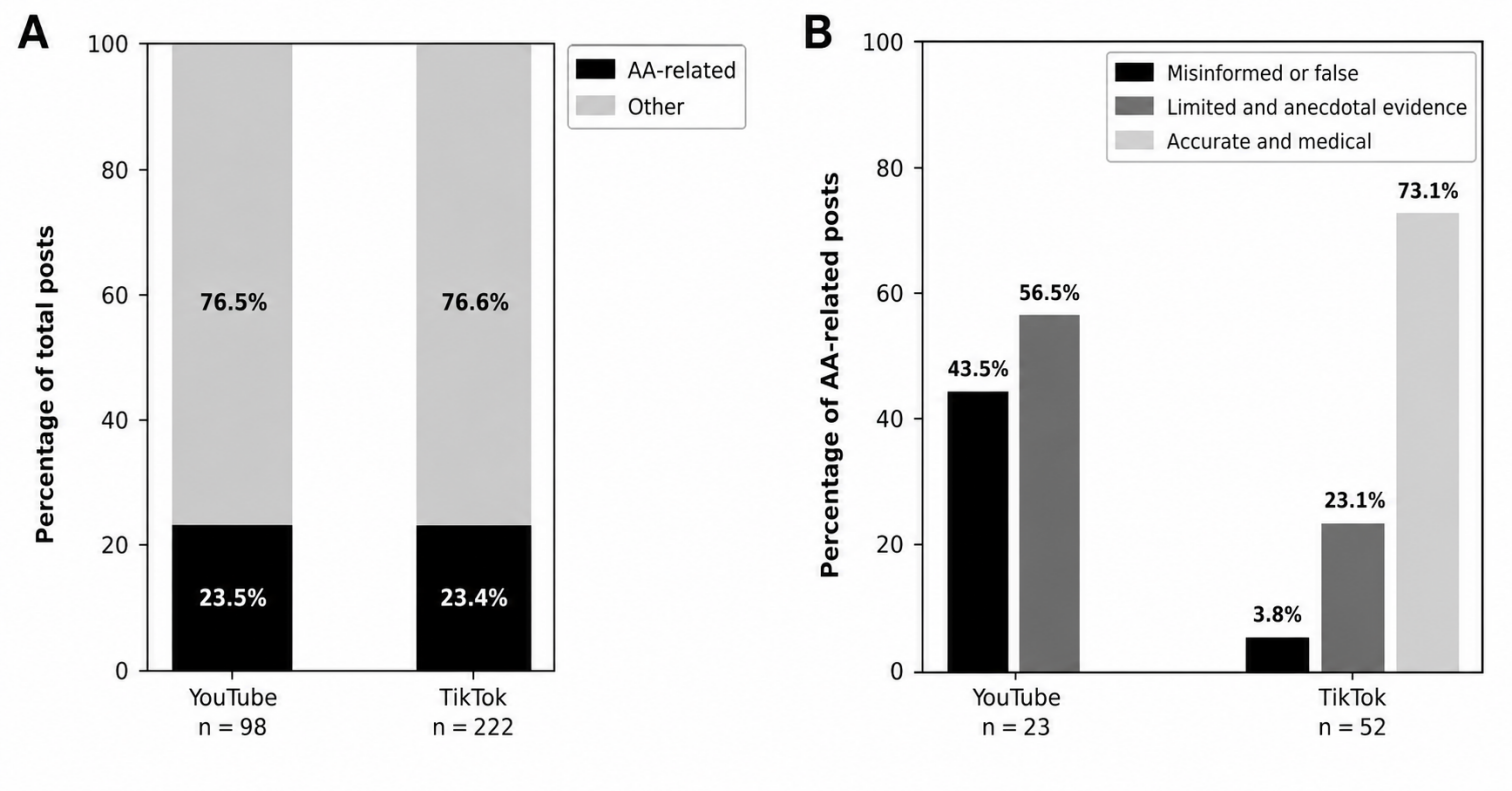
(A) Distribution of 98 YouTube videos and 222 TikTok posts tagged with #alopecia and/or #alopeciatreatment, showing the proportion that contained alopecia areata (AA) treatment–related content vs other content. (B) Distribution of 23 YouTube videos and 52 TikTok posts specifically addressing AA treatment, showing the proportions categorized as misinformed and/or false, limited or anecdotal, and accurate or medical.

Statistical analysis revealed a statistically significant difference in the proportion of accurate content between the YouTube videos categorized as “accurate and medical data–backed” and those classified as “limited or anecdotal evidence” (*P*=.002). No videos in this sample were categorized as “misinformed and/or false” (Figure 1).

**Table 1.** Standard alopecia areata treatments recommended by the American Academy of Dermatology [10].

Condition	Treatment options	Details
1 or 2 bald spots (<1 year)	Wait-and-see approach, corticosteroids, and minoxidil (Rogaine)	Many patients experience spontaneous regrowth. Corticosteroids may stimulate regrowth, while minoxidil may help maintain hair growth.
Children (aged ≤10 years)	Corticosteroids and minoxidil	Corticosteroids are often applied daily. Minoxidil may help maintain regrowth.
Children (aged >10 years)	Contact immunotherapy and JAK^a^ inhibitors	Contact immunotherapy typically involves weekly treatments. JAK inhibitors may promote regrowth in severe cases.
Patchy hair loss	Corticosteroid injections, topical corticosteroids, anthralin, and minoxidil	Corticosteroid injections may be effective but painful. Topical therapies may support regrowth.
Extensive hair loss	Contact immunotherapy, JAK inhibitors, and other systemic medications	Systemic therapies and immunotherapy may promote significant regrowth in severe disease.
Hiding hair loss^b^	Styling products; wigs, hairpieces, or scalp prostheses; shaving; eyebrow powder; artificial eyebrows; and microblading	Cosmetic approaches may help improve appearance and confidence.

a JAK: Janus kinase.

b Supportive or cosmetic management approaches rather than disease-directed alopecia areata treatments.

### TikTok Content Analysis

A total of 222 TikTok videos related to #alopecia and #alopeciatreatment were reviewed. Of these, 76% (n=170) were classified as “other.” The remaining 23% (n=52) of TikTok posts focused on AA treatment. Of these, 73% (n=38) were categorized as “accurate and medical data–backed,” while 23% (n=12) were classified as “limited or anecdotal evidence.” A small portion of posts (n=2, 3%) were identified as “misinformed and/or false,” and these posts included content promoting unsubstantiated treatments, such as the use of unapproved topical solutions or unproven dietary supplements.

Although TikTok demonstrated a higher prevalence of misinformation-related content, there was no statistically significant difference between platforms (*P*=.15; Figure 1B).

## Discussion

Our study found that a large portion of videos obtained through #alopecia and #alopeciatreatment searches were unrelated to AA treatment or instead focused on other forms of alopecia. Among videos specifically discussing AA, explicit misinformation was limited, although some TikTok videos contained misleading or non–evidence-based treatment recommendations. Additionally, many posts emphasized emotional support and community-building themes, highlighting both the informational and psychosocial roles social media plays for patients with AA.

AA is a visual and psychosociological disruptive disease that may prompt patients to seek online resources outside of the patient-physician relationship. Social media has become a ubiquitous resource for patients to search for health care–related information, including treatments for AA. It is critical that physicians and health care professionals understand the extent and depth of the information that their patients are exposed to on social media to be aware of potential misinformation dissemination and provide correct educational materials on treatment. Previous studies have only sought to understand social media post myths related to alopecia, including untrue hair loss causes and understudied alopecia treatments [5].

The results of this study highlight that a sizeable portion of the video posts centered on #alopecia or #alopeciatreatment searches were related to other types of alopecia (traction alopecia, alopecia universalis, etc), were not understandable, or were unrelated to the treatment of AA. Among the videos related to AA, none of the YouTube videos contained overt misinformation, while 2 TikTok videos included misinformation. However, videos with limited or anecdotal information could prove misleading to patients because social media influencers may be selling their own products or *oils* for profit as accepted treatments along with the standardized treatments such as minoxidil. Although posts containing misinformation represented a minor portion of the dataset, highly viewed or widely disseminated content may still disproportionately influence patient perceptions and treatment choices.

Given that most of the videos were not specifically about AA, another potential pitfall is patients following guidance on another type of alopecia altogether, which would not be appropriate for AA. The results indicate that patients may seek other forms of hair loss solutions that could further stagnate or worsen their hair loss, including the use of wigs, which can damage the hairline, and other oils, which could hamper the growth of hair. Without appropriate guidance on how to use these hair loss supportive measures, patients can worsen their hair loss or cause other diseases, such as scarring alopecia (traction alopecia), related to their scalp health.

There were limitations to the study, including the exclusion of nonvideo social media platforms such as Reddit, which could offer additional insights. The limited hashtag searches could have been broadened to include tags such as #alopeciacure, providing a wider range of posts and enabling a more thorough understanding of the information patients access. Content categorization was performed by a single reviewer, and interrater reliability statistics were not calculated, which may limit reproducibility and introduce potential classification bias. The physician creator inclusion criteria may also limit broader generalizability across international and multidisciplinary social media content environments.

Engagement metrics such as views, likes, comments, shares, and follower counts were not included in our analysis, which limited assessment of the overall reach and potential influence of misinformation-related content. Additionally, this study was limited by the relatively short 1-week data collection period, which may not fully capture the fluctuating nature of social media content and the algorithms of the TikTok and YouTube platforms. Content visibility and viewer engagement may rapidly evolve over time, and the trends observed in AA treatment discussions may have differed over a longer collection period.

Future studies should explore whether patients with AA follow their physician’s treatment guidance or turn to social media influencer advice due to barriers in accessing health care or mistrust in the patient-physician relationship. Future studies may further evaluate how creator characteristics and audience engagement influence the spread and impact of AA content on social media. According to Gümüş et al [11], 96% of patients reported that they would trust their physician’s advice if it conflicted with social media content on AA and would try to reach their physician through online medical platforms. Gantenbein et al [12] further confirm that the information patients find online does not significantly disrupt their relationship with their physicians. However, the same study also underscores the need for physicians to engage actively on social media to ensure that patients receive correct information upfront.

Our study found that patients both provided and sought out community-building support through video posts, highlighting the importance of physicians addressing the emotional impacts of AA rather than just the physical symptoms. As Aldhouse et al [13] demonstrated, patients often feel sadness, helplessness, social isolation, and a desire for support during treatment, which aligns with the emotional content seen in many posts from our study. It is vital for physicians to adopt a holistic approach in treating AA, offering support for emotional struggles and connecting patients to communities of others experiencing similar challenges.

Health care professionals can counteract misinformation by creating clear and educational social media content centered on evidence-based AA treatments. Meah et al [14] further support the use of evidence-based therapies for AA, including intralesional corticosteroids, topical treatments, and systemic therapies commonly used in clinical practice. These therapies may vary based on disease severity, patient population, and individual treatment considerations, nuances that may not always be fully reflected in social media discussions surrounding AA treatment. Creating precise, informative materials such as digital flyers that can be distributed in clinics gives patients straightforward explanations of their disease and treatment options, supporting ongoing education even after their visit.

As AA remains a hair loss disease of great physical concern and social limitation to patients, it is timely for healthcare stakeholders to ensure that the social media platforms are used as tools of proactive education to combat any misinformation related to the disease.

## Abbreviations

AA: alopecia areata
